# Aeropalynological analysis of airborne pollen in Posof Türkiye and its relationship with meteorological factors

**DOI:** 10.1038/s41598-025-05867-4

**Published:** 2025-07-12

**Authors:** Melih Karabağ, Salih Akpınar

**Affiliations:** Department of Biology, Faculty of Science and Letters, Kafkas University, Kars, Turkey

**Keywords:** Airborne pollen, Pollen diversity, Posof, Türkiye, Volumetric trap, Ecology, Plant sciences

## Abstract

This study investigated airborne pollen concentrations in the Posof District of Ardahan Province during 2020 and 2021. The significance of the study lies in the region’s location on the Georgian border and its transitional position between the Eastern Anatolia and Black Sea regions, which influence its vegetation and pollen composition. Pollen sampling was conducted using Hirst-type volumetric 7-day pollen and spore trap, identifying 39 taxa-21 woody and 18 herbaceous. The dominant pollen taxa were Pinaceae (37.18%), *Betula* (14.55%), Poaceae (9.35%), Cupressaceae/Taxaceae (7.26%), and Urticaceae (7.04%). The highest pollen concentrations were recorded in May, indicating a seasonal peak. Taxonomic pollen diversity in Posof showed similarities to both Black Sea and Eastern Anatolian regions. Spearman’s correlation analysis revealed a positive correlation between mean daily temperature and wind speed with pollen concentrations, whereas relative humidity showed a negative correlation. Due to the region’s geographical and climatic conditions, the onset dates of identified pollen types were delayed by 2–4 weeks compared to similar studies. This delay is attributed to altitude and specific meteorological conditions, particularly lower spring temperatures, persistent snow cover, and delayed warming, which collectively postpone plant phenology and pollen release. The findings contribute to understanding airborne pollen dynamics in Posof and provide valuable data for regional aerobiological studies.

**Supplementary Information:**

The online version contains supplementary material available at 10.1038/s41598-025-05867-4.

## Introduction

Atmospheric pollen refers to pollen grains suspended in the air and transported primarily by wind, originating from anemophilous plant species. These particles significantly affect public health, particularly among individuals with pollen allergies. Pollen grains from genera such as *Betula* (birch) and Poaceae (grasses) are recognized as major aeroallergens that contribute to allergic rhinitis and asthma^1–3^. The size of airborne pollen grains, typically ranging between 20 and 45 μm, influences their allergenic potential and their depth into the respiratory tract^4–6^. Smaller pollen grains or fragmented particles can reach the lower airways, intensifying allergic symptoms^6,7^.

Pollen allergies, commonly referred to as hay fever or allergic rhinitis, affect a substantial portion of the global population, particularly in Europe. Symptoms include sneezing, nasal congestion, itchy eyes, and in some cases, food-related cross-reactions with specific pollen types^8–10^. Recent studies highlight the potential role of climate change in prolonging pollen seasons and increasing allergenic pollen production^11^. However, it is important to note that phenological responses to climate change are species-specific and may differ across regions and taxa^12^.

Understanding the dynamics of atmospheric pollen-its seasonal patterns, diversity, and intensity-is essential for managing allergic disease burden and informing public health policies^13^. Pollen concentrations are influenced by several interrelated factors including local flora, meteorological conditions, and geographical characteristics. Variables such as temperature, precipitation, relative humidity, and wind speed are particularly important in shaping flowering periods, pollen release, and atmospheric transport^1,3,14–18^. Moreover, the spatial distribution of pollen-producing vegetation contributes to regional differences in airborne pollen loads^19^.

The abundance and diversity of atmospheric pollen are not solely determined by the presence of flowering plants, but also by their phenological behavior and ecological interactions with environmental variables^20^. The composition of the regional flora, particularly the dominance of anemophilous species, plays a key role in determining the airborne pollen spectrum^21,22^. Meteorological conditions can either promote or inhibit pollen release and dispersion. For instance, elevated temperatures and dry weather often accelerate flowering and pollen emission, while rainfall can reduce airborne pollen by washing it out of the atmosphere^20^. In transitional zones such as Posof, where floristic elements from both Euro-Siberian and Irano-Turanian phytogeographic regions coexist, these climatic and ecological interactions result in a complex and dynamic airborne pollen profile^23,24^.

Located in Eastern Anatolia, Türkiye, Posof experiences a unique blend of continental and maritime (Black Sea) climatic influences due to its geographic position. This convergence of climate types supports rich and diverse vegetation. Despite this, no comprehensive aerobiological studies have previously been conducted in the region. Given its proximity to the Georgian border and its location within a shared phytogeographic transition zone, Posof also shows ecological similarities with adjacent areas of Georgia. Although limited, existing aerobiological studies from Tbilisi, Kutaisi and Western Georgia have reported seasonal pollen patterns and dominant taxa-such as Cupressaceae, *Platanus*, *Morus*, *Ulmus*, and *Artemisia*-that are also common in Northeastern Türkiye. These observations highlight the need for cross-border studies to better understand regional pollen dynamics and potential allergenic exposures^25–27^.

Based on these considerations, this study hypothesizes that the airborne pollen composition and its seasonal variation in Posof are shaped by the region’s phytogeographical complexity and climatic variability. The main objectives of the study are: (i) to identify and classify the airborne pollen taxa recorded in Posof during the 2020–2021 period; (ii) to determine the timing and duration of the main pollen seasons (MPS) for taxa with a relative contribution exceeding 3% to Annual Pollen Integral (APIn); (iii) to analyze the relationships between dominant pollen taxa and key meteorological factors; and (iv) to identify high-risk periods for allergic individuals based on the temporal distribution of allergenic taxa.

## Materials and methods

### Study area, flora and climate

**Fig. 1:**
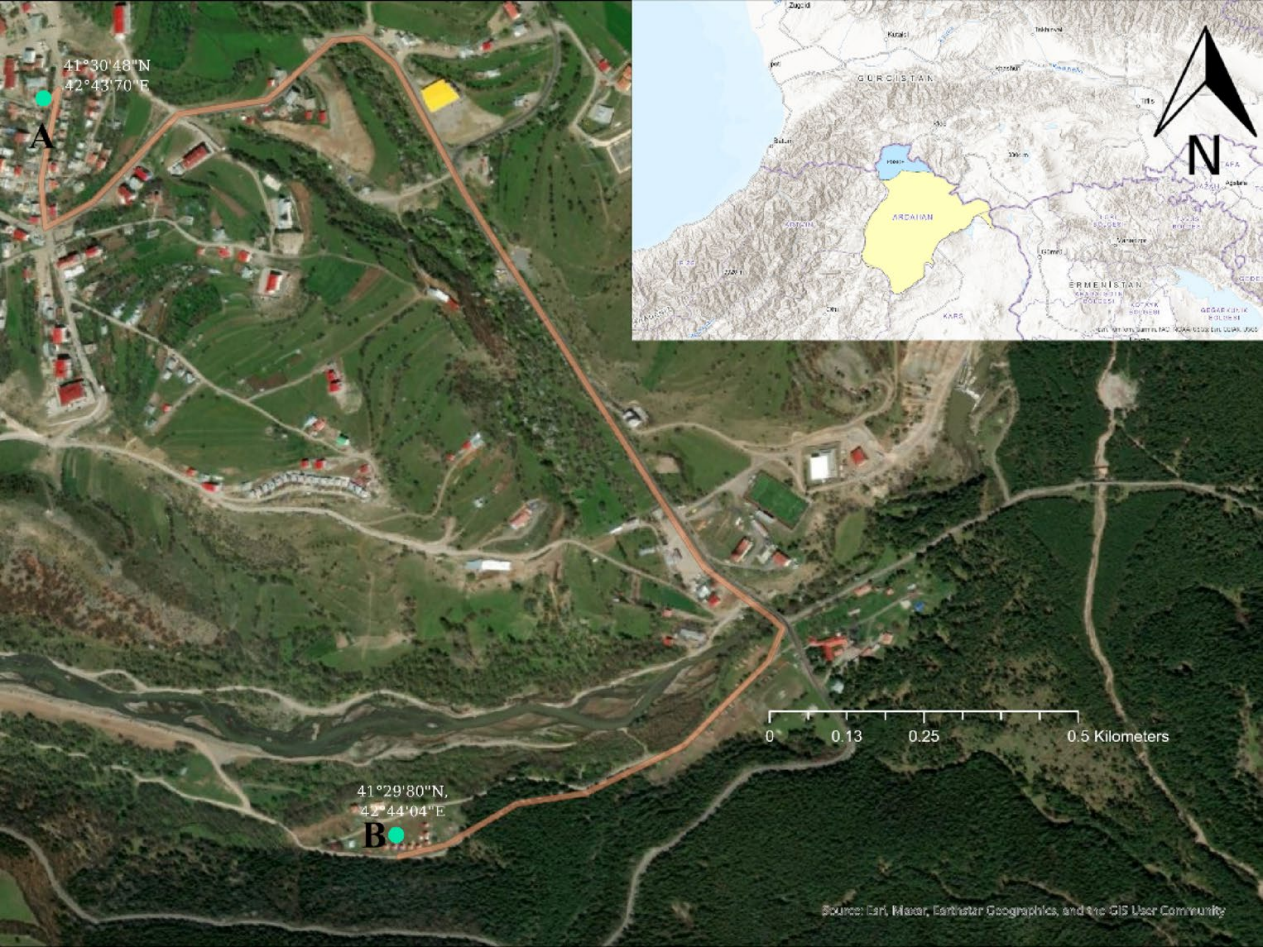
Location map of Posof (**A**) sampling station, (**B**) weather station.

Posof, located in the Eastern Anatolia Region of Türkiye, lies on the border with the Republic of Georgia and is positioned at 41°30′42″N, 42°43′45″E (Fig. 1). The district covers an area of 623 km^2^ and has an average elevation of 1583 m. It shares a 77 km-long border with Georgia, specifically with the Autonomous Republic of Adjara to the north. Posof is bordered by Georgia to the north and northeast, Şavşat district of Artvin Province to the east and southeast, Damal district to the south, and Hanak district to the west.

The region supports rich floristic diversity due to its transitional location between the Euro-Siberian and Irano-Turanian phytogeographic regions, as well as its topographic complexity and unique climate. It is situated within a natural forest area dominated by species such as *Quercus* (oak), *Pinus* (pine), *Abies* (fir), *Larix* (larch), *Fagus* (beech), *Carpinus* (hornbeam), *Betula* (birch), and *Corylus* (hazelnut). In 1994, Demirkuş and Erik identified 750 taxa in the region, comprising 333 genera, 720 species, 18 subspecies, and 12 varieties across 82 families^28^. In a more recent study in 2010, Esen documented 1225 taxa, belonging to 411 genera and 95 families, across the districts of Posof, Hanak, and Damal^29^. Among these, 9 taxa were gymnosperms and 1200 were angiosperms, classified under spermatophytes. Many of the genera, species, and subspecies reported in these floristic surveys correspond to the dominant pollen taxa identified in the atmosphere of Posof. Table S1 presents the plant diversity of dominant airborne pollen taxa in the region.

**Fig. 2:**
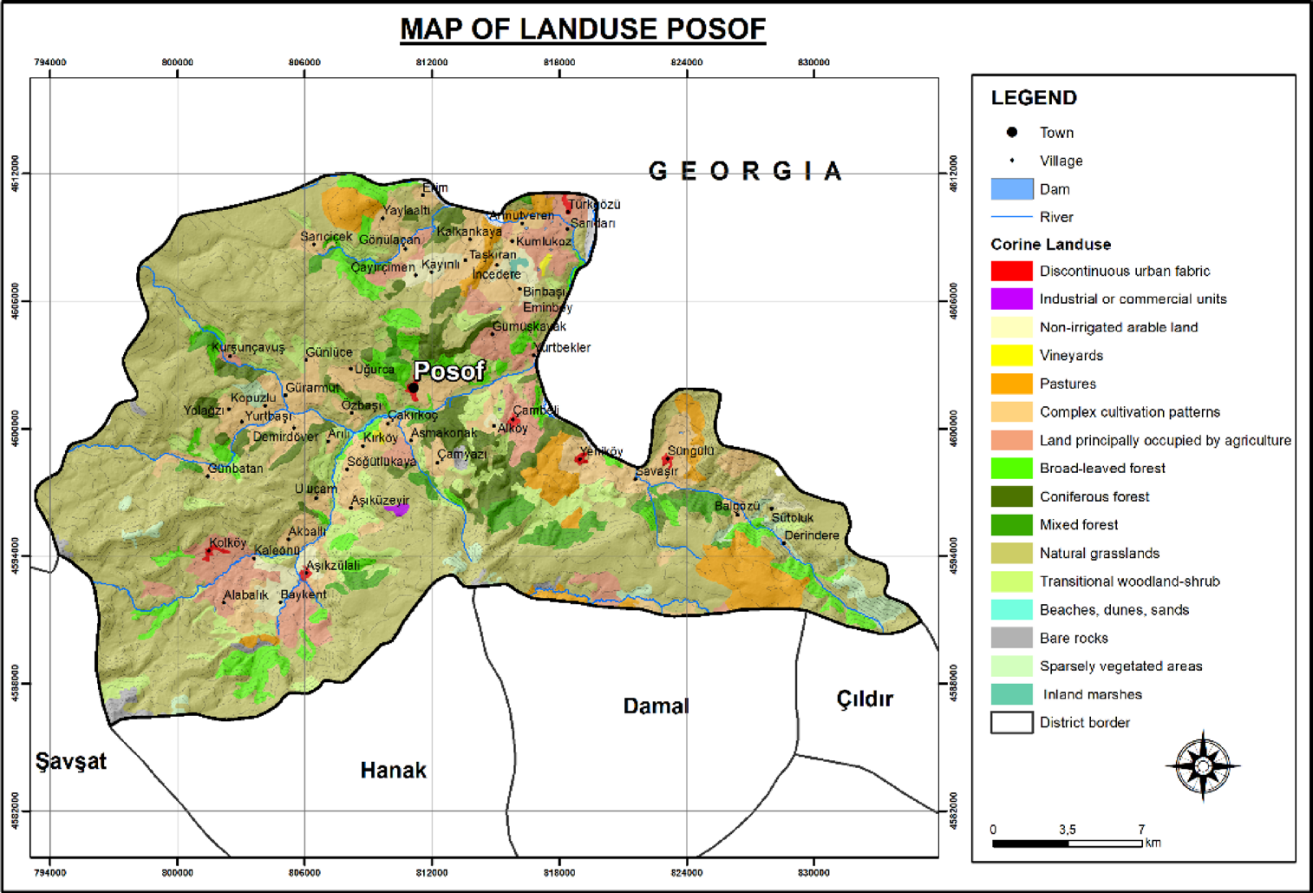
Map of land use of Posof.

To provide ecological context for airborne pollen composition, land cover data were obtained from the CORINE (Coordination of Information on the Environment) Land Cover 2018 dataset. This classification was used to identify dominant vegetation types within the study area. According to CORINE analysis, pastures (5.07%) and non-irrigated arable lands (1.90%) were among the primary vegetation types associated with herbaceous pollen sources, particularly Poaceae. In addition, several natural and semi-natural vegetation types were identified, including natural grasslands (51.38%), which typically support a wide range of Poaceae and other herbaceous taxa; mixed agricultural areas (12.06%), which combine croplands with semi-natural vegetation; and agricultural areas with natural vegetation (6.63%), which contain both cultivated species and native ruderal herbs (Fig. 2).

Forest formations were also prominent: broad-leaved forests (5.76%) were dominated by genera such as *Quercus*, *Fagus*, and *Carpinus*; coniferous forests (4.57%) were primarily composed of *Pinus* and *Picea*; and mixed forests (2.99%) included both coniferous and deciduous elements. Transitional woodland-shrub areas (6.36%) contained early successional woody taxa and shrubs such as *Betula*, *Salix*, and *Corylus*. Lastly, sparsely vegetated areas (1.77%) were characterized by stress-tolerant low vegetation, including species from the Asteraceae and Amaranthaceae families. These land cover categories contribute to the diversity and seasonal dynamics of airborne pollen and offer a landscape-level understanding of pollen source vegetation in the region (Fig. 2).

**Fig. 3:**
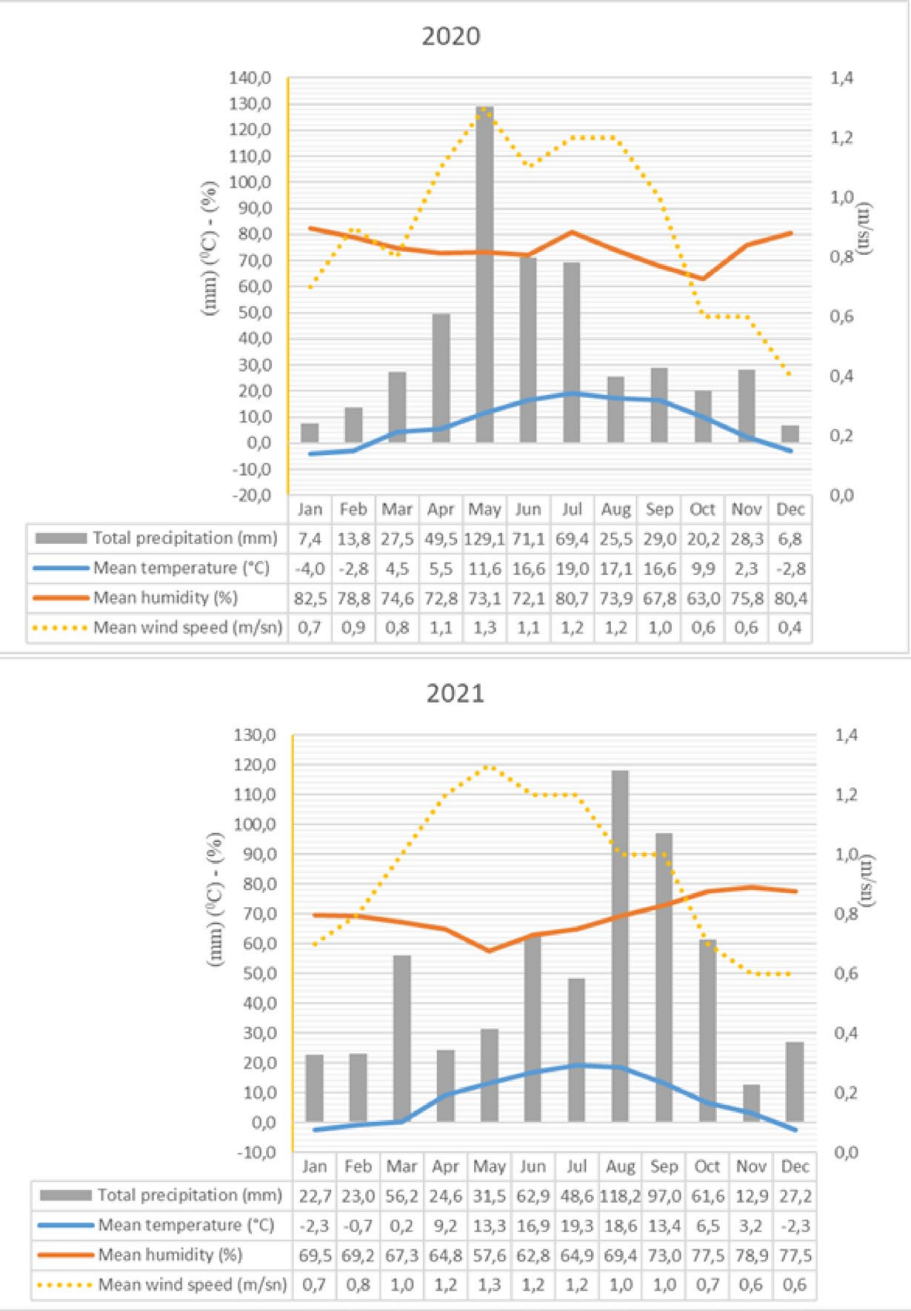
Monthly variation in mean temperature, mean relative humidity, total precipitation and mean wind speed in Posof for 2020–2021.

**Fig. 4:**
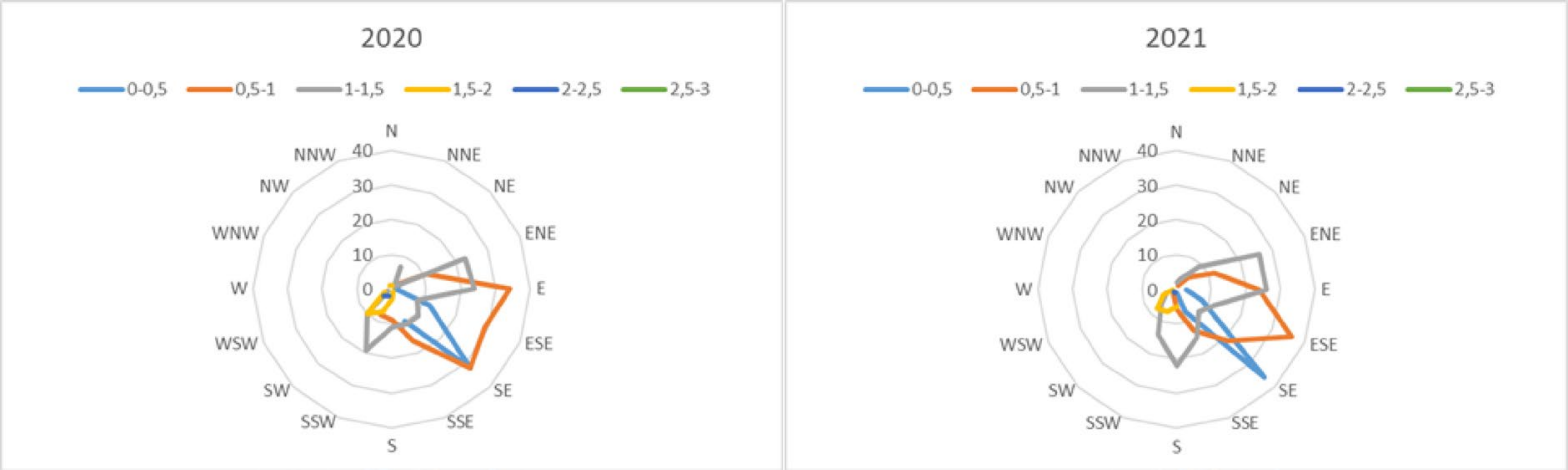
Wind rose 2020–2021.

The climate of the study area is continental, characterized by cold, snowy winters and short, hot, and rainy summers. Winter temperatures frequently fall below freezing while summer temperatures may reach up to 30 °C due to the district’s elevation. Based on 50-year meteorological data from the Turkish State Meteorological Service, January was the coldest month (-11.2 °C), and August was the warmest (24.7 °C). August also received the highest solar radiation, with an average of 8.2 h of sunshine per day. May was the wettest month, with 16.8 rainy days and 93.8 mm of precipitation, while January was the driest, with 19.88 mm. Meteorological parameters for the study years-such as relative humidity, temperature, precipitation, wind speed, and wind direction-were summarized in Figs. 3 and 4.

### Aerobiological method and statistical analysis

During the 2020–2021 study period, airborne pollen sampling was carried out in Posof using a Hirst-type volumetric pollen and spore trap (Lanzoni VPPS 2010). The device was installed at a height of 15 m on the rooftop of a central building (41°30′30″N, 42°43′42″E) (Fig. 1). Weekly samples were sectioned into 7 equal parts (48 mm each) in the laboratory to obtain daily preparations. The daily and hourly average pollen concentrations were determined by examining samples under a Leica light microscope at 400X magnification. Pollen grains were counted at 2 mm intervals, corresponding to 1-hour segments. Results were expressed as pollen/m^3^ following the recommendations of the Spanish Aerobiological Network (REA) and international guidelines^30^.

Daily variations and MPS were determined using the 2.5-97.5% method proposed by Andersen^31^. The beginning of the MPS was defined as the day when daily pollen concentrations reached 2.5% of the Annual Pollen Integral (APIn), and the end corresponded to 97.5% of the accumulated value. This method is widely accepted for its reliability and allows standardized comparisons across taxa and years. Threshold values for daily pollen concentrations affecting sensitive vary according to different sources^30,32,33^. Threshold values for allergenic pollen concentrations affecting sensitive individuals were adopted from the REA and are presented in Table 1^30^. According to these thresholds, low concentrations may only affect highly sensitive individuals, moderate levels impact a significant proportion of allergic patients, and high concentrations can trigger symptoms in most allergic individuals regardless of sensitivity. The number of moderate and high-risk days was used to estimate allergy risk periods.

**Table 1:** Pollen threshold values of sensitive individuals and the number of days at risk for Posof^21^.

Group	Taxa	Total daily pollen amount	2020	2021
Group 1	Urticaceae	Nil: < 1 grain/m^3^		
		Low: 1–15 grains/m^3^	86 days	89 days
		Moderate: 16–30 grains/m^3^	19 days	14 days
		High: >30 grains/m^3^	14 days	16 days
Group 2	Poaceae	Nil: < 1 grain/m^3^		
		Low: 1–25 grains/m^3^	123 days	140 days
		Moderate: 26–50 grains/m^3^	26 days	10 days
		High: >50 grains/m^3^	14 days	2 days
Group 3	*Betula*	Nil: < 1 grain/m^3^		
		Low: 1–30 grains/m^3^	79 days	85 days
		Moderate: 31–50 grains/m^3^	11 days	10 days
		High: >50 grains/m^3^	14 days	7 days
Group 4	Pinaceae	Nil: < 1 grain/m^3^		
		Low: 1–50 grains/m^3^	47 days	116 days
		Moderate: 51–200 grains/m^3^	14 days	10 days
		High: >200 grains/m^3^	17 days	10 days
	Cupressaceae/Taxaceae	Nil: < 1 grain/m^3^		
		Low: 1–50 grains/m^3^	97 days	93 days
		Moderate: 51–200 grains/m^3^	11 days	2 days
		High: >200 grains/m^3^	1 days	

The use of percentage-based criteria in aerobiological studies offers significant methodological advantages. It minimizes the influence of sporadic pollen grains that may appear outside the main season, ensures consistency in season delimitation, and allows for standardized comparisons across different regions, taxa, and years^34^. In this study, percentage values were also used to compare dominant pollen taxa with other regions instead of absolute pollen counts, in order to reduce the impact of regional environmental differences and sampling variations. This approach is particularly effective in retrospective phenological assessments and long-term monitoring studies^30,31^.

Pollen concentrations were correlated with meteorological parameters such as temperature, wind speed, relative humidity, and total precipitation for 2020–2021 obtained from the Turkish State Meteorological Service, located approximately 1.3 km away from the sampling device (41°29′49″N, 42°44′04″E) (Fig. 1). The relationship between meteorological factors and pollen concentrations was evaluated using Spearman’s correlation test in SPSS 20, with significance set at *p* < 0.05.

## Results

Over the two-year study in Posof, APIn 42,587 pollen*day/m^3^ were recorded, representing 39 taxa. In 2020, an APIn of 25,711 pollen*day/m^3^ from 39 taxa were observed, while in 2021, 16,876 pollen*day/m^3^ from 35 taxa were recorded. In addition to identified taxa, a small proportion of pollen grains (0.37%) could not be taxonomically assigned due to insufficient morphological characteristics. Although these grains were clearly identified as pollen, they were categorized as “Unidentified” in Table 2. The pollen of Ericaceae, *Cistus*, *Olea*, and *Xanthium* taxa, which were detected in 2020, were not identified in 2021. It was determined that woody pollen was more abundant in the atmosphere than herbaceous pollen in both years. Specifically, the most abundant woody pollen included Pinaceae, *Betula*, Cupressaceae/Taxaceae, *Quercus*, *Populus*, *Alnus*, *Fagus*, *Fraxinus*, *Morus*, and *Carpinus*. On the other hand, the most commonly observed herbaceous pollen were Poaceae, Urticaceae, *Artemisia*, *Rumex*, and Amaranthaceae (Tables 2 and 3).

**Table 2:** Annual pollen integral (APIn) (pollen*day/m^3^ and percentage of pollen taxa recorded in Posof atmosphere (2020–2021).

Taxa	2020		2021		Mean	
	APIn (pollen*day/m^3^)	%	APIn (pollen*day/m^3^)	%	APIn (pollen*day/m^3^)	%
Pinaceae	9916	38.57	5919	35.07	7918	37.18
***Betula***	**3778**	**14.69**	**2418**	**14.33**	**3098**	**14.55**
**Cupressaceae/Taxaceae**	**1891**	**7.35**	**1199**	**7.10**	**1545**	**7.26**
**Quercus**	**819**	**3.19**	**363**	**2.15**	**591**	**2.78**
***Populus***	**285**	**1.11**	**791**	**4.69**	**538**	**2.53**
***Alnus***	**652**	**2.54**	**388**	**2.30**	**520**	**2.44**
***Fagus***	**263**	**1.02**	**363**	**2.15**	**313**	**1.47**
***Fraxinus***	**282**	**1.10**	**337**	**2.00**	**310**	**1.45**
***Morus***	**292**	**1.14**	**185**	**1.10**	**239**	**1.12**
***Carpinus***	**160**	**0.62**	**292**	**1.73**	**226**	**1.06**
*Salix*	90	0.35	239	1.42	165	0.77
*Juglans*	96	0.37	109	0.65	103	0.48
Rosaceae	107	0.42	75	0.44	91	0.43
*Ulmus*	52	0.20	46	0.27	49	0.23
*Corylus*	81	0.32	8	0.05	45	0.21
*Acer*	15	0.06	10	0.06	13	0.06
*Olea*	11	0.04	-	-	6	0.03
*Liqustrum*	7	0.03	3	0.02	5	0.02
*Tilia*	5	0.02	2	0.01	4	0.02
*Cistus*	4	0.02	-	-	2	0.01
Ericaceae	2	0.01	-	-	1	0.00
**Woody taxa**	**18,808**	**73.15**	**12,747**	**75.53**	**15,778**	**74.10**
**Poaceae**	**2807**	**10.92**	**1174**	**6.96**	**1991**	**9.35**
**Urticaceae**	**1491**	**5.80**	**1507**	**8.93**	**1499**	**7.04**
***Artemisia***	**756**	**2.94**	**324**	**1.92**	**540**	**2.54**
***Rumex***	**336**	**1.31**	**223**	**1.32**	**280**	**1.31**
**Amaranthaceae**	**229**	**0.89**	**226**	**1.34**	**228**	**1.07**
Boraginaceae	244	0.95	146	0.87	195	0.92
*Ambrosia*	259	1.01	124	0.73	192	0.90
*Plantago*	226	0.88	92	0.55	159	0.75
Fabaceae	136	0.53	88	0.52	112	0.53
Apiaceae	85	0.33	10	0.06	48	0.22
Asteraceae	56	0.22	37	0.22	47	0.22
Lamiaceae	47	0.18	27	0.16	37	0.17
Cyperaceae	29	0.11	42	0.25	36	0.17
Brassicaceae	40	0.16	6	0.04	23	0.11
Caryophyllaceae	21	0.08	18	0.11	20	0.09
Cannabaceae	25	0.10	12	0.07	19	0.09
*Taraxacum*	8	0.03	19	0.11	14	0.06
*Xanthium*	3	0.01	-	-	2	0.01
**Herbaceous taxa**	**6798**	**26.44**	**4075**	**24.15**	**5437**	**25.53**
Unidentified	105	0.41	54	0.32	80	0.37
**Total**	**25,711**	**100.00**	**16,876**	**100.00**	**21,294**	**100.00**

**Table 3:** Pollen grains in the Posof atmosphere (%), based on the two-year average data.

Taxa	Jan	Feb	Mar	Apr	May	Jun	Jul	Aug	Sep	Oct	Nov	Dec	Total
Pinaceae	0.03	0.03	0.03	0.11	20.68	16.16	0.11	0.03					37.18
*Betula*		0.23	3.69	4.42	6.00	0.21							14.55
Cupress./Tax.	0.02	0.05	0.11	1.84	4.38	0.85	0.01						7.26
*Quercus*				0.24	2.42	0.12							2.78
*Populus*			0.34	2.15	0.03								2.53
*Alnus*		1.18	1.01	0.19	0.06								2.44
*Fagus*			0.002	0.32	1.13	0.02							1.47
*Fraxinus*			0.07	0.48	0.89	0.01							1.45
*Morus*				0.39	0.15	0.57	0.005						1.12
*Carpinus*		0.03	0.12	0.81	0.11								1.06
*Salix*			0.01	0.52	0.20	0.03	0.002						0.77
*Juglans*				0.08	0.40								0.48
Rosaceae				0.04	0.33	0.05							0.43
*Ulmus*			0.04	0.16	0.03								0.23
*Corylus*		0.01	0.05	0.06	0.08								0.21
*Acer*				0.04	0.01								0.06
*Olea*					0.01	0.02							0.03
*Liqustrum*				0.005	0.01	0.01							0.02
*Tilia*						0.002	0.01						0.02
*Cistus*						0.01							0.01
Ericaceae						0.005							0.005
**Woody taxa**	0.05	1.53	5.49	11.87	36.91	18.07	0.15	0.03					74.10
Poaceae			0.01	0.78	0.71	4.03	2.71	0.85	0.23	0.02			9.35
Urticaceae			0.005	0.08	0.07	3.25	2.77	0.83	0.04	0.01			7.04
*Artemisia*						0.03	0.52	1.29	0.63	0.06			2.54
*Rumex*				0.08	0.77	0.29	0.17	0.002					1.31
Amaranthaceae					0.02	0.18	0.19	0.37	0.27	0.04			1.07
Boraginaceae					0.04	0.69	0.16	0.02	0.002				0.92
*Ambrosia*								0.43	0.47				0.90
*Plantago*				0.005	0.06	0.35	0.24	0.05	0.04				0.75
Fabaceae					0.06	0.31	0.15	0.01					0.53
Apiaceae				0.04	0.02	0.08	0.07	0.02					0.22
Asteraceae			0.002	0.01	0.04	0.08	0.06	0.03	0.002				0.22
Lamiaceae					0.02	0.06	0.08	0.02					0.17
Cyperaceae				0.02	0.11	0.04							0.17
Brassicaceae					0.08	0.03							0.11
Caryophyllaceae					0.02	0.06	0.01						0.09
Cannabaceae						0.04	0.03	0.02					0.09
*Taraxacum*						0.03	0.01	0.01	0.002				0.06
*Xanthium*									0.002	0.005			0.01
**Herbaceous taxa**			0.02	1.01	2.01	9.53	7.17	3.97	1.70	0.13			25.53
Unidentified	0.01	0.01	0.02	0.09	0.09	0.05	0.04	0.03	0.03	0.01	0.002		0.37
**Total**	0.05	1.54	5.52	12.98	39.01	27.65	7.36	4.02	1.73	0.14	0.002		100.00

**Fig. 5:**
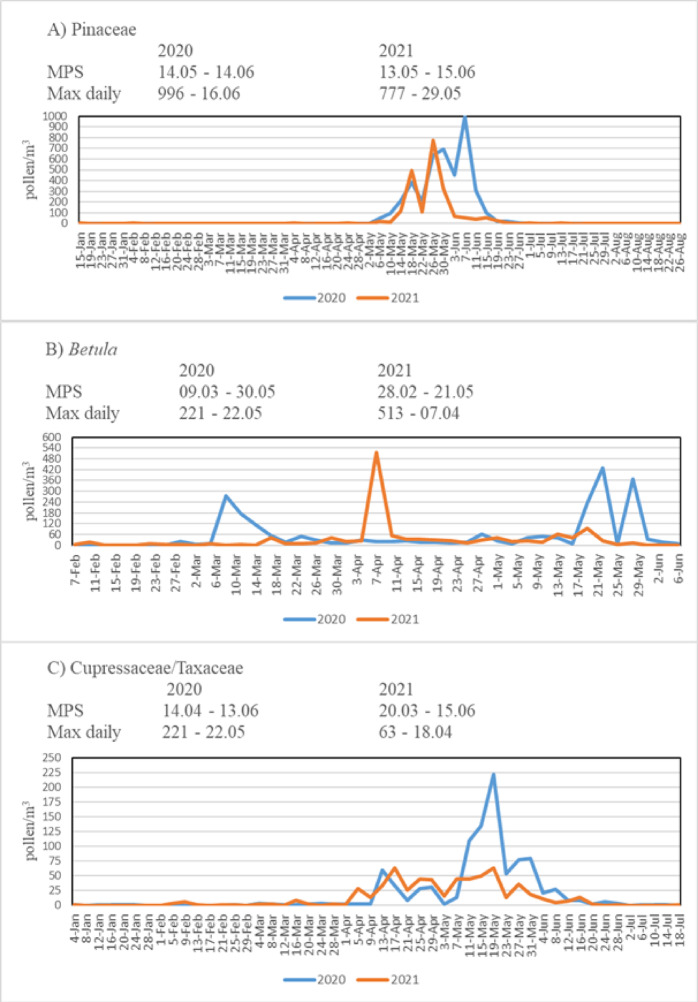
Daily pollen concentration of three most dominant woody taxa for the atmosphere of Posof (**A** Pinaceae,** B** *Betula*,** C** CupressaceaeTaxaceae).

**Fig. 6:**
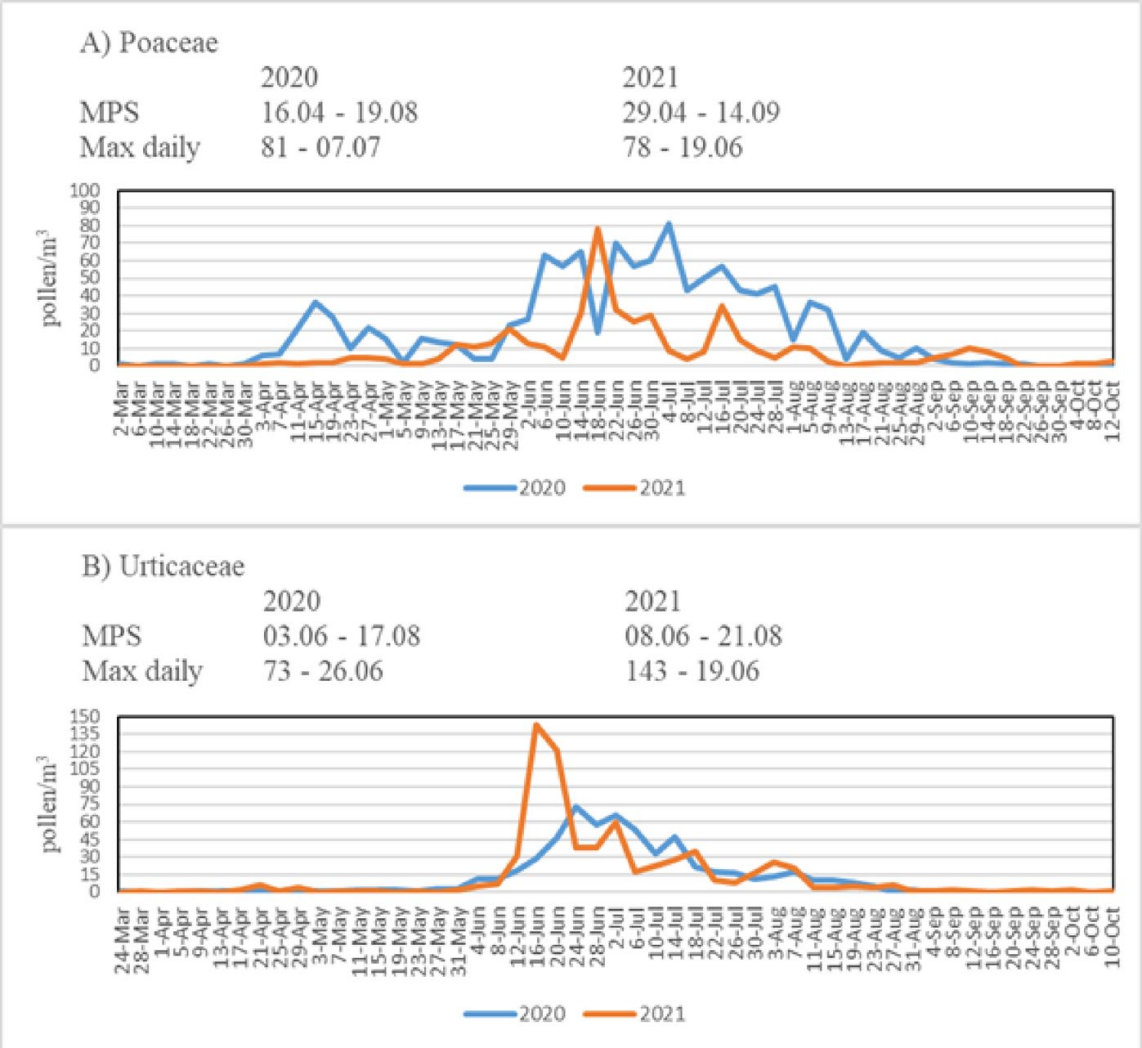
Daily pollen concentration of two most dominant herbaceous taxa for the atmosphere of Posof (**A** Poaceae,** B** Urticaceae).

In both years, the highest pollen concentrations were observed from March to July. Interestingly, in the first year, only Cupressaceae/Taxaceae pollen were detected in January, whereas in the second year, Pinaceae pollen were also present. Woody pollen were identified between January and July, while herbaceous pollen were observed from March to October. No pollen was recorded in November and December due to temperatures dropping below 4 °C. Although pollen diversity varied by month, the highest diversity was observed in May (29 taxa in 2020, 28 taxa in 2021), which also corresponded to the highest overall concentrations. According to daily pollen data, a significant increase in concentrations was observed in May and June in 2020, and from April to June in 2021. The increase in April is attributed to *Betula*, Cupressaceae/Taxaceae, and *Populus*, while the rise in May and June is due to Pinaceae, *Betula*, Cupressaceae/Taxaceae, Urticaceae, and Poaceae (Figs. 5 and 6). The highest concentrations were observed in May, accounting for 35.33% in 2020 and 44.62% in 2021. Woody pollen peaked in May, whereas herbaceous pollen peaked in June.

A total of 15 pollen taxa with a relative abundance exceeding 1% together accounted for 93.14% of the total pollen composition. These taxa were identified as follows: Pinaceae (37.18%), *Betula* (14.55%), Poaceae (9.35%), Cupressaceae/Taxaceae (7.26%), Urticaceae (7.04%), *Quercus* (2.78%), *Artemisia* (2.54%), *Populus* (2.53%), *Alnus* (2.44%), *Fagus* (1.47%), *Fraxinus* (1.45%), *Rumex* (1.31%), *Morus* (1.12%), Amaranthaceae (1.07%), and *Carpinus* (1.06%) (Table 2). The first taxa detected in the atmosphere of Posof were woody taxa including Cupressaceae/Taxaceae, Pinaceae, *Alnus*, *Betula*, and *Carpinus*. Herbaceous taxa such as *Artemisia*, *Ambrosia*, and *Xanthium* were first identified in the atmosphere from late June onwards (Table 2). Detailed information on MPS, daily fluctuations, and maximum daily concentration (max daily) for the woody taxa Pinaceae, *Betula* and Cupressaceae/Taxaceae (with relative abundances exceeding 3%) is presented in Fig. 5, while data for the herbaceous taxa Poaceae and Urticaceae, which also exceeded 3% in relative abundance, is shown in Fig. 6.

The total number of low medium, and high risk days in Table 1 represents the number of days within a year when pollen is observed, while the sum of medium and high days indicates the number of days exceeding allergenic threshold levels for sensitive individuals. These five dominant taxa constituted 77.33% of the total pollen in 2020 and %72.39% in 2021. When evaluating the MPS, the durations for *Betula*, Cupressaceae/Taxaceae, and Urticaceae ranged between 60 and 87 days, with durations of 82, 60, and 75 days in 2020 and 82, 87, and 74 days in 2021, respectively. Poaceae showed the longest MPS with 125 days in 2020 and 138 days in 2021. The MPS of Pinaceae despite being one of the most abundant taxa, was relatively short, lasting 31 days in 2020 and 33 days in 2021, typically between mid-May and mid-June (Figs. 5 and 6).

Spearman’s correlation test (*p* < 0.05) was performed to determine the statistical relationships between daily pollen concentrations and meteorological variables, including daily mean temperature, wind speed, total precipitation, and relative humidity. Stronger correlations were observed between pollen concentrations and temperature and humidity (*p* < 0.05). Significant positive correlations were identified between daily mean temperature and the concentrations of *Alnus*, Amaranthaceae, *Artemisia*, *Betula*, Cupressaceae/Taxaceae, *Fraxinus*, Pinaceae, Poaceae, *Populus*, *Rumex*, and Urticaceae. Negative correlations were found between relative humidity and the concentrations of *Artemisia*, *Betula*, Cupressaceae/Taxaceae, *Fraxinus*, Pinaceae, Poaceae, *Populus*, *Quercus*, and *Rumex*, while Urticaceae showed a positive correlation. No significant correlation was observed between total precipitation and any taxon, but wind speed showed positive correlations with the concentrations of *Artemisia*, *Betula*, Cupressaceae/Taxaceae, *Fraxinus*, Pinaceae, Poaceae, and *Quercus* (Table 4).

**Table 4:** Results of spearman’s correlation analysis and results (*p < 0.05).

Taxa	Mean daily temperature	Mean daily relative humidity	Mean daily precipitation	Mean daily wind speed
*Alnus*	**0.376** ^*^	− 0.122	− 0.130	− 0.140
Amaranthaceae	**0.329** ^*^	− 0.118	− 0.141	0.006
*Artemisia*	**0.432** ^*^	**− 0.200** ^*^	− 0.126	**0.404** ^*^
*Betula*	**0.421** ^*^	**− 0.341** ^*^	− 0.126	**0.270** ^*^
*Carpinus*	0.112	− 0.168	− 0.285	0.188
Cupres./Tax.	**0.345** ^*^	**− 0.555** ^*^	− 0.111	**0.470** ^*^
*Fagus*	0.053	− 0.164	− 0.100	0.034
*Fraxinus*	**0.463** ^*^	**− 0.398** ^*^	− 0.127	**0.409** ^*^
*Morus*	− 0.080	− 0.171	− 0.119	− 0.222
Pinaceae	**0.270** ^*^	**− 0.420** ^*^	0.028	**0.275** ^*^
Poaceae	**0.526** ^*^	**− 0.268** ^*^	− 0.138	**0.189** ^*^
*Populus*	**0.354** ^*^	**− 0.371** ^*^	− 0.020	0.252
*Quercus*	0.210	**− 0.345** ^*^	− 0.112	**0.269** ^*^
*Rumex*	0.016	**− 0.320** ^*^	− 0.096	0.098
Urticaceae	**0.646** ^*^	**0.180** ^*^	− 0.014	− 0.011

## Discussion

This study provides a comprehensive evaluation of atmospheric pollen diversity in Posof, emphasizing its distinct geographical and climatic characteristics. Posof, located at the intersection of the Euro−Siberian and Irano-Turanian phytogeographic regions, exhibits a unique pollen profile influenced by its transitional climate and mountainous geography. The findings highlight the biological and allergenic properties of dominant taxa, the influence of meteorological factors, and comparisons with similar studies conducted in Türkiye and globally.

Similar studies in Türkiye and worlwide were selected based on comparable altitude, climate characteristics, and pollen diversity (Table 5)^15,35–46^. In Türkiye, regions with continental climates (e.g., Kars^41^, Kars-Sarıkamış^42^, Van^39^, Mardin^35^, Gümüşhane^15^, Elazığ^38^, and Konya^40^) were compared with maritime regions such as Trabzon^36^ and Sinop^37^. The international aerobiological studies included in Table 5 were selected to allow ecological comparison with the Posof region by considering multiple environmental parameters, including climate type, elevation, and dominant pollen composition. For example, Vigo (Spain) has a humid oceanic climate, Mexico City a subtropical highland climate (2240 m), and Funchal (Portugal) a mild Mediterranean climate^47–49^. Although these sites differ climatically from Posof, they were included due to their similarity in dominant pollen taxa and seasonal concentration trends. In contrast, Bratislava (Slovakia) has a temperate continental climate, which is more comparable to Posof both in terms of climate regime and seasonal temperature variation^50^. While Posof is situated at approximately 1500 m, the inclusion of Mexico City, also located at high altitude, enables elevation-based comparisons. This selection strategy, combining both ecologically similar and contrasting regions, provides a broader framework to interpret the influence of environmental factors on airborne pollen diversity and dynamics.

**Table 5:** Dominant taxa in similar studies in Türkiye and the world.

Sampling sites	APIn (pollen*day/m^3^)	Woody (%)	Herbaceous (%)	Pinaceae (%)	Betula (%)	Poaceae (%)	Cupressaceae/ Taxaceae (%)	Urticaceae (%)
Posof	21,294	74.1	25.53	37.18	14.55	9.35	7.26	7.04
Mardin^35^ (Türkiye)	3857	62.66	36.86	3.84	0.36	21.21	27.79	2.32
Trabzon^36^ (Türkiye)	29,150	69.78	30.00	11.59	5.91	13.56	19.29	7.10
Sinop^37^ (Türkiye)	46,707	69.50	30.50	16.10	1.30	17.00	13.70	
Gümüşhane^15^ (Türkiye)	20,772	85.60	14.40	30.92	4.68	8.80	17.73	0.47
Elazığ^38^ (Türkiye)	108,313	76.39	23.18	20.16	0.13	9.10	19.26	4.92
Van^39^ (Türkiye)	4163	58.20	41.80	2.94	0.72	20.94	10.53	2.79
Konya^40^ (Türkiye)	4343	61.29	36.34	29.36	2.72	16.09	8.29	1.50
Kars^41^ (Türkiye)	14,936	36.34	63.57	18.29	1.47	36.74	2.01	5.91
Kars-Sarıkamış^42^ (Türkiye)	18,955	36.34	63.56	29.79	0.55	44.60	2.54	2.33
Vigo^43^ (Spain)	3150	54.20	45.70	25.10	3.60	21.10	2.10	14.60
Bratislava^44^ (Slovakia)	36,608	65.00	35.00	6.28	23.38	5.11	9.93	18.84
Mexico City^45^ (Mexico)	14,367	–	–	4.95	–	2.43	17.70	1.29
Funchal^46^ (Portugal)	–	52.72	44.64	9.07	3.52	16.02	13.61	20.64

An average of 21,294 pollen grains was detected annually in the Posof atmosphere. Compared to surrounding regions, Posof exhibited higher APIn values than Gümüşhane^15^, Van^39^, Kars^41^, Mardin^35^, and was closely aligned with values recorded in Trabzon^36^ but lower than in Sinop^37^ and Elazığ^38^ (Table 5). A total of 39 taxa were identified, including 21 woody and 18 herbaceous taxa, with 15 dominant taxa comprising 93.14% of the total pollen composition. Although Posof is located in a transitional zone between the Euro-Siberian and Irano-Turanian phytogeographical regions and is therefore expected to exhibit high botanical diversity, the number of identified pollen taxa (39) falls within the range reported in other aerobiological studies conducted in Türkiye and abroad. For example, comparable pollen diversity was recorded in Kars^41^ (39), Elazığ^38^ (38), Van^39^ (35), Konya^40^ (35), and Tbilisi^25^ (34), while slightly higher diversity was noted in Mardin^35^ (44), Trabzon^36^ (45), and Kars-Sarıkamış^42^ (43). In more coastal or floristically heterogeneous areas, such as Sinop^37^ (61) and Gümüşhane^15^ (70), higher pollen diversity was observed, likely due to broader ecological gradients and milder climates. Internationally, similar diversity levels were reported in Mexico City^45^ (42), Funchal (42), and Bratislava^44^ (34), whereas Vigo^43^ showed notably higher richness (73 taxa). Therefore, the pollen diversity observed in Posof reflects regional aerobiological characteristics, climatic conditions, and the dominance of anemophilous taxa, rather than the full floristic potential of the territory. Similar patterns were observed in regions like Mardin^35^ (62.66%), Sinop^37^ (69.5%), Trabzon^36^ (69.78%), Van^39^ (58.2%), Elazığ^38^ (76.39%), Vigo^43^ (54.2%), Bratislava^44^ (65%), and Mexico City^45^ (70%), where woody taxa dominated due to climatic and geographic factors (Table 5). Posof’s microclimate, shaped by surrounding mountains, supports rainy winters and warm summers, blending continental and Black Sea climates. Consequently, atmospheric pollen reflects traits of both regions. In addition to Türkiye and other international studies, relevant aerobiological research conducted in Georgia-Posof’s direct eastern neighbor-also offers valuable insights. For example, atmospheric pollen monitoring in Tbilisi and Kutaisi^25^ (2016) identified Cupressaceae (43.6%), *Platanus* (10.3%), *Morus* (6.5%), *Ulmus* (5.9%), *Artemisia* (4.3%), and *Populus* (3.9%) as dominant taxa, many of which were also prominent in Posof. Similarly, the five-year pollen calendar from Tbilisi^26^ (2013–2017) reported comparable seasonal trends, with extended spring seasons for Poaceae and Asteraceae. A modern pollen-vegetation study in Southern Georgia^27^ (2004) also confirmed the dominance of Amaranthaceae, *Artemisia*, *Quercus*, and *Fagus* along an altitudinal transect, reflecting steppe to forest transitions that parallel ecological gradients in the Posof region. These transboundary similarities support the idea of shared phytogeographic patterns and suggest a wider regional coherence in airborne pollen composition and phenology.

A clear interannual variation was observed between the 2020 and 2021 pollen seasons, with APIn being considerably higher in 2020 (25711 pollen*day/m^3^) than in 2021 (16876 pollen*day/m^3^). Interestingly, this higher pollen load occurred despite 2020 being characterized by cooler temperatures and higher spring precipitation, which would typically be expected to delay or reduce pollen release. In contrast, 2021 featured warmer and drier conditions during April-May, yet recorded lower overall pollen values. This suggests that pollen dynamics are influenced not only by current meteorological conditions but also by cumulative ecological and phenological factors, including plant development from the previous year, soil moisture storage, and species-specific flowering responses. Additionally, increased wind speeds in 2021 may have contributed to higher peak concentrations for certain taxa despite the overall lower annual total. These findings highlight the complex interplay between climate variability and pollen production, emphasizing the need for multi year data interpret atmospheric pollen trends reliably^30,51^.

In mediterranean climates, peak pollination occurs in March-April, whereas in colder regions like Posof, it peaks in May-June^16,41,42,52,53^. As observed in others studies from Türkiye and Europe, herbaceous taxa dominate in summer, while woody taxa dominate in spring^15,35,37,54–59^. This trend is associated with early-blooming taxa, including Pinaceae, Cupressaceae, *Betula*, *Populus*, Poaceae, *Rumex*, and Urticaceae^39,60^.

Rather than directly assigning taxa to specific phytogeographical regions, it is more appropriate to consider their prevalence in regions examined by previous aerobiological studies. For instance, families such as Urticaceae and *Betula* were also dominant in studies conducted in the Euro-Siberian influenced areas such as Sinop^37^, Trabzon^36^, and Bratislava^44^. In contrast, higher frequencies of Poaceae and Pinaceae were reported in more continental or Irano-Turanian influenced regions such as Kars^41^, Elazığ^38^, Van^39^, and Mardin^35^. These comparisons provide a more empirical basis for understanding floristic similarities, while avoiding overgeneralization, as most families comprise species that are widely distributed and phenologically diverse. The annual pollen concentrations of Pinaceae in Posof was comparableto that reported for cities such as Konya^40^ and Ankara^61^ but higher than Elazığ^38^, Kars^41^, and Kars-Sarıkamış^42^. However, it was lower than that of Aydın-Didim^62^ and Kastamonu^3^. Pinaceae pollen, mostly observed in May and June, is a significant cause of spring allergies^63^. Common species in the region include *Abies nordmanniana* subsp. *nordmanniana*, *Picea orientalis*, and *Pinus sylvestris* var. *Hamata*^28,29^. Recent studies indicate that rising temperatures and changing precipitation patterns are altering the timing and duration of pollen seasons across various plant taxa, including Pinaceae^64,65^. These changes may lead to earlier pollen release and potentially shorter overall pollen seasons, as trees adapt to new climatic conditions. Although the MPS of Pinaceae family is relatively short, the presence of days with medium to high threshold levels on almost all days when it was detected in Posof indicates a significant risk of allergy. From an allergy perspective, other important taxa were *Betula*, Cupressaceae/Taxaceae, Poaceae, *Populus*, and Urticaceae. Although *Populus* pollen is generally less abundant than other allergenic taxa, several studies have reported its allergenic potential, particularly in individuals sensitized to tree pollens. Recent research has identified specific allergenic proteins such as Pop n 2 in *Populus nigra*, confirming its relevance as a respiratory allergen^66^.

The dominance of pollen from coniferous taxa such as Pinaceae and broad-leaved deciduous taxa such as *Betula* and *Populus* aligns well with the general vegetation structure of the Posof region. According to CORINE land cover data for the Posof region, coniferous forests account for approximately 4.57% of the area, while broad-leaved forests represent about 5.76%. This spatial distribution supports the observed pollen composition, as these vegetation types are prominent pollen sources in the area. Incorporating such vegetation data helps validate the pollen results and emphasizes the ecological relevance of the detected airborne taxa.

In the study area, *Betula pendula* Both, *Betula litwinowii* Doluch., and *Betula recurvata* (Ig. Vassil.) V. Vassil. are present, and *Betula* forests cover extensive areas^28,29^. Among these taxa, *Betula* pollen is particularly noteworthy due to both its abundance and allergenic potential. In Northern and Central Europe, *Betula* pollen is recognized as a major cause of allergic rhinoconjunctivitis and asthma^67–69^. In Europe, between 6.4% and 22.4% of the population is sensitized to *Betula* pollen, while in Türkiye, sensitization rates range from 2.3 to 7.79% in children and from 3.81 to 49.7% in adults^70–72^. The considerable amounts of *Betula* pollen observed at high altitudes in Posof are thus of both ecological and public health interest. Its abundance in the region also reflects the influence of Black Sea flora, as *Betula* thrives under similar climatic conditions.

Cupressaceae family, which is widely distributed globally, comprises 140 species belonging to 15 genera. In Türkiye, 8 species belonging to the genera *Cupressus* and *Juniperus* are naturally distributed, while Taxaceae family is represented by a single species, *Taxus baccata*. In the region, there are sub-taxa belonging to the genera *Juniperus*^73^. Members of the Cupressaceae/Taxaceae family are the main cause of seasonal respiratory allergies such as allergic rhinitis and allergic asthma, particularly during the winter period in Mediterranean countries^1,74,75^. However, due to the very low temperatures during winter months, Cupressaceae/Taxaceae pollen, like other taxa, were recorded at high levels in the atmosphere during spring. Despite the long duration of the pollen season, their allergenic capacity is limited, as also demonstrated by previous studies showing that Cupressaceae pollen grains, although abundant, release sub-pollen particles with minimal IgE reactivity. The number of days exceeding the threshold values was only 12 in 2020 and 2 in 2021.

Poaceae pollen, accounting for 0.35% of the total concentrations, is the most abundant herbaceous pollen in Posof and a prominent allergen globally^1,76,77^. In Posof, as in other cities of the Eastern Anatolian region Poaceae pollen was the most abundant herbaceous pollen, but the pollen season was not as long as in those regions^38,39,41,42^. This relatively shorter season may be associated with the spatial distribution of Poaceae-dominated areas around the sampling site. According to CORINE land cover data, pastures (5.07%) and non-irrigated arable lands (1.90%) represent the main grass-related vegetation types in the region. While these habitats are sufficient to support high Poaceae pollen concentrations, their limited extent compared to more agriculturally intensive regions may contribute to a more condensed pollen season in Posof.

Another commonly observed taxa in the atmosphere is the Urticaceae family, which includes the genera *Urtica* and *Parietaria*, both naturally distributed in the region^29^. Due to the high similarity of their pollen under the microscope, these taxa were not identified at the genera level in the current study. Urticaceae pollen was recorded from March to October, similar to Poaceae. However, the main pollen season for Urticaceae was much shorter compared to Poaceae. MPS of Urticaceae has lasted very briefly compared to Vigo^43^ and Funchal City^46^. Although the flowering period of Urticaceae species is extended, the true pollen release period, defined as the main pollen season, is relatively short due to the limited number of Urticaceae species in the region. This results in a shorter and more synchronized peak pollen release. However, the approximately 30 risk days per year for allergy patients, combined with the widespread presence of highly allergenic Parietearia plants, may pose significant issues for Posof.

Taxa with less than 3% concentration (e.g., *Quercus*, *Artemisia*, *Populus*, *Alnus*, *Fagus*, *Fraxinus*, *Rumex*, Amaranthaceae, and *Carpinus*) collectively contribute 17.86% of the total pollen but do not significantly affect allergy risk.

This study explored the atmospheric pollen diversity in Posof and its relationship with meteorological factors. Rising temperatures were associated with earlier pollen release for woody taxa like *Betula* and Pinaceae, consistent with findings in Bursa^78^, Gümüşhane^15^, and Trabzon^36^. High levels of precipitation can reduce pollen concentration by shortening the time pollen remains airborne^79,80^. However, precipitation can also affect plant growth and pollen production. While adequate rainfall promotes pollen production, excessive rainfall can cause pollen to settle on the ground^15^. Precipitation reduced airborne pollen concentrations but promoted vegetation growth, explaining Posof’s higher Annual Pollen Integral (APIn) compared to drier regions like Mardin^35^, Van^39^, and Kars-Sarıkamış^42^. High wind speeds facilitate the widespread dispersion of pollen, while low wind speeds result in localized pollen concentrations^81^. Wind direction also plays a role in pollen distribution, with winds from specific directions increasing pollen density^82^. Wind speed enhanced the dispersion of Poaceae and Cupressaceae, consistent with findings from Mediterranean and continental climates, including regions like İzmir – Çeşme^83^, Kars^41^, and Hatay^84^. High humidity can shorten the time pollen remains airborne and cause pollen grains to rupture^85^. Ruptured pollen grains release allergenic proteins, increasing the risk of allergic reactions. The effects of humidity in Posof, which show a negative correlation with the pollen concentrations of most taxa, are similar to those reported in Van^39^ (Cupressaceae/Taxaceae, *Fraxinus*, *Populus*) and Kars^41^ (*Artemisia*, Cupressaceae/Taxaceae, Pinaceae, Poaceae, *Quercus*, Urticaceae). However, they contrast with the positive correlations reported in Sinop^37^, Karabük^86^, and Mersin^16^ suggesting regional variations in pollen-humidity interactions.

To further support the interpretation of meteorological influences, wind direction data and CORINE land cover mapping were integrated into the analysis. The prevailing spring winds were predominantly from the south and southwest, and coniferous forest zones—particularly dense in these directions—align with the elevated concentrations of Pinaceae pollen. This spatial relationship, combined with topographical features of Posof, helps clarify the likely origin zones of dominant airborne taxa. The inclusion of vegetation cartography and wind patterns thus strengthens the ecological relevance of the pollen data and supports a spatially informed understanding of pollen transport in the region.

## Conclusions

This study presents the first comprehensive aerobiological assessment of the Posof district, revealing that airborne pollen diversity in the region is strongly influenced by its unique biogeographical location, topographical complexity, and transitional climate. Dominated by woody taxa, the pollen composition reflects the floristic convergence between the Euro-Siberian and Irano-Turanian phytogeographic regions.

During the two-year sampling period, 21,294 pollen grains were recorded, representing 39 taxa—21 woody and 18 herbaceous. Among these, five dominant taxa (Pinaceae, Betula, Cupressaceae/Taxaceae, Poaceae, and Urticaceae) constituted over 70% of the total pollen load and contributed significantly to allergenic risk. A total of 90 and 66 allergenic days were identified in 2020 and 2021, respectively, primarily between May and July. This period poses the highest risk to sensitive individuals due to both high pollen loads and overlapping main pollen seasons (MPS).

Meteorological parameters, particularly temperature and relative humidity, were found to significantly influence daily pollen concentrations. High wind speeds enhanced pollen dispersion, while rainfall reduced airborne concentrations but supported vegetation growth. The incorporation of wind direction data and CORINE land cover maps strengthened the spatial interpretation of pollen sources. For instance, the predominance of coniferous forests in southern and southwestern sectors aligns with prevailing wind patterns and high Pinaceae pollen levels.

Land cover analysis revealed that natural grasslands, pastures, and non-irrigated arable lands—key sources of Poaceae pollen—are limited in extent, which likely contributes to the condensed Poaceae pollen season observed in Posof compared to other regions in Eastern Anatolia. Similarly, sparsely vegetated zones and mixed forests support other allergenic taxa such as Artemisia and Cupressaceae.

Cross-border comparison with Georgian regions such as Tbilisi and Kutaisi revealed similar dominant taxa and pollen seasonality, supporting the ecological continuity between northeastern Türkiye and the Caucasus. These transboundary similarities suggest a broader regional pattern of airborne pollen composition that may inform shared allergenic risk assessments.

From a public health perspective, the results provide critical data for allergists and healthcare professionals. The identification of MPS, pollen concentration peaks, and allergenic threshold exceedance days offers a foundation for region-specific pollen forecasts, allergy advisories, and long-term monitoring programs.

## Electronic supplementary material

Below is the link to the electronic supplementary material.

Supplementary Material 1

## Data Availability

The datasets generated during and/or analysed during the current study are available from the corresponding author on reasonable request.
