# Supplementary material for: Aeropalynological analysis of airborne pollen in Posof Türkiye and its relationship with meteorological factors
Source: Sci Rep. 2025 Jul 12;15:25271. doi: 10.1038/s41598-025-05867-4 (PMC12255752; doi:10.1038/s41598-025-05867-4)
Supplement: Supplementary file 1 — Supplementary Material 1 [file 41598_2025_5867_MOESM1_ESM.docx]

**Table S1.** Plant diversity in the posof region of dominant pollen detected in the atmosphere^29^

| **Dominant pollen taxa** | **Plant diversity** |
| --- | --- |
| ***Alnus*** | *Alnus glutinosa* (L.) Gaertner subsp. *glutinosa* |
| **Amaranthaceae** | *Amaranthus retroflexus* L., *Atriplex nitens* Schkuhr., *Chenopodium foliosum* (Moench.) Aschers., *Chenopodium botrys* L., *Chenopodium album* L. subsp. *album* var. *Album* |
| ***Artemisia*** | *Artemisia marschaliana* Sprengel, *Artemisia vulgaris* L., *Artemisia austriaca* Jacq., *Artemisia absinthium* L., *Artemisia splendens* Willd., *Artemisia chamaemelifolia* Vill. |
| ***Betula*:** | *Betula litwinowii* Doluch., *Betula pendula* Roth., *Betula recurvata* (Ig. Vassil.) V. Vassil., |
| ***Carpinus*** | *Carpinus betulus* L. |
| **Cupressaceae/Taxaceae** | *Juniperus communis* L. var. *Communis,* *Juniperus communis* L. var. *saxatilis* Pall., *Juniperus communis* L. subsp. *hemisphaerica* (Presl) Nyman, *Juniperus oblonga* Bieb., *Juniperus oxycedrus* L. subsp. *oxycedrus,* *Juniperus sabina* L. |
| ***Fagus*** | *Fagus orientalis* Lipsky |
| ***Fraxinus*** | *Fraxinus angustifolia* Vahl subsp. *oxycarpa* (Bieb. ex Willd.) Franco ex Rocha, *Fraxinus excelsior* L. subsp. *coriariifolia* (Scheele) E. Murray |
| ***Morus*** | *Morus rubra* L. |
| **Pinaceae** | *Abies nordmanniana* (Stev.) Spach subsp. *nordmanniana*, *Picea orientalis* (L.) Link, *Pinus sylvestris* var. *hamata* Steven |
| **Poaceae** | *Agropyron* Gaertner, *Agrostis* L., *Alopecurus* L., *Avena* L., *Bothriochloa* O. Kuntze, *Brachypodium* L., *Briza* L., *Bromus* L., *Calamagrostis* Adanson, *Cynodon* L., *Cynosurus* L., *Dactylis* L., *Deschampsia* P. Beauv., *Elymus* L., *Eremopoa* Roshev., *Festuca* L., *Gaudiniopsis* Eig, *Glyceria* R. Br., *Hordeum* L., *Lolium* L., *Melica* L., *Milium* L., *Phleum* L., *Poa* L., *Setaria* P. Beauv.*Stipa* L., *Trisetum* Pers, *Zea* L. |
| ***Populus*** | *Populus tremula* L., *Populus nigra* L. subsp. *nigra* |
| ***Quercus*** | *Quercus macranthera* Fisch. et Mey. ex Hohen. subsp. *syspirensis* (C. Koch) Menitsky, *Quercus petraea* (Mattuschka) Liebl. subsp. *iberica* (Steven ex Bieb.) Krassiln, *Quercus petraea* (Mattuschka) Liebl. subsp. *pinnatiloba* (C. Koch) Menitsky, *Quercus vulcanica* (Boiss. et Heldr. ex) Kotschy, |
| ***Rumex*** | *Rumex acetosella* L., *Rumex scutatus* L. Dk., *Rumex tuberosus* L. subsp. *horizontalis* (Koch) Reich |
| **Urticaceae** | *Urtica urens* L., *Urtica dioica* L., *Parietaria lusitanica* L. |
